# Survey of electron density changes in the daytime ionosphere over the Arecibo Observatory due to lightning and solar flares

**DOI:** 10.1038/s41598-021-89662-x

**Published:** 2021-05-13

**Authors:** Caitano L. da Silva, Sophia D. Salazar, Christiano G. M. Brum, Pedrina Terra

**Affiliations:** 1 Department of Physics and Langmuir Lab, New Mexico Tech, Socorro, NM USA; 2 Arecibo Observatory and University of Central Florida, Arecibo, PR USA

**Keywords:** Atmospheric science, Plasma physics, Natural hazards, Space physics

## Abstract

Optical observations of transient luminous events and remote-sensing of the lower ionosphere with low-frequency radio waves have demonstrated that thunderstorms and lightning can have substantial impacts in the nighttime ionospheric D region. However, it remains a challenge to quantify such effects in the daytime lower ionosphere. The wealth of electron density data acquired over the years by the Arecibo Observatory incoherent scatter radar (ISR) with high vertical spatial resolution (300-m in the present study), combined with its tropical location in a region of high lightning activity, indicate a potentially transformative pathway to address this issue. Through a systematic survey, we show that daytime sudden electron density changes registered by Arecibo’s ISR during thunderstorm times are on average different than the ones happening during fair weather conditions (driven by other external factors). These changes typically correspond to electron density depletions in the D and E region. The survey also shows that these disturbances are different than the ones associated with solar flares, which tend to have longer duration and most often correspond to an increase in the local electron density content.

## Introduction

The lower ionosphere marks the separation between neutral and ionized regions of the Earth’s atmosphere, and has been colloquially referred to as the *edge of space*. The Earth and the ionosphere create a cavity that traps low-frequency electromagnetic waves, making long-range radio communications possible. Leveraging this phenomenology, the high variability of the lower ionosphere, the so-called ionospheric D region, has been extensively probed with ELF/VLF remote sensing^1–5^. The processes that control the ion-electron production in the lower ionosphere are mostly dominated by the precipitation of energetic particle flux from the radiation belts, photoionization from solar UV radiation and hard X-rays during daytime, meteor ablation, solar and cosmic ray precipitation, etc.^6^ Each one of these ionization processes has a different weight in the formation of the D region, depending on the geomagnetic latitude and season. During the daytime, the D region is largely maintained by Lyman-$\alpha$ radiation (121.6 nm) between 70 and 80 km altitude, and by solar X-ray flux (0.1–1 nm) between 80 and 90 km altitude. Impulsive surges on solar radiation, known as solar flares, thus create sudden enhancements on the electronic content of this region^7^. Very-low frequency (VLF) remote sensing shows that solar flares produce a lowering of the effective VLF reflection height roughly in proportion to the logarithm of the X-ray flare intensity from a typical mid-day unperturbed value of about 71 km down to about 58 km for the strongest flares^8^.

However, there is a growing body of evidence that not only forcing from above, such as solar flares, can create sudden alterations in the lower ionosphere^1,2,9–17^. A number of authors have showed that underlying thunderstorms can also modify the lower ionosphere due to the penetration of thunderstorm^10^ and lightning^9,11–16^ quasi-electrostatic fields causing electron heating and electron-impact ionization, or due to thunderstorm-originated gravity waves that can modulate the local electron density^17,18^. Here, we look for potential evidence of the former mechanism. A byproduct of thunderstorms, transient luminous events^13^ (TLEs), have appeared as an opportunity for optical remote-sensing of the lower ionosphere-mesosphere system, by being not only a consequence of the electrical coupling of atmospheric regions, but also its thermometer^19^. However, both TLE observations in the optical range and remote-sensing of the lower ionosphere with low-frequency radio waves are not well-suited for providing quantitative estimates of the electron density changes in the ionospheric D region due to lightning, especially during daytime (due to the excess ionization and optical radiation caused by sunlight). So here enters the Arecibo Observatory Incoherent Scatter Radar (ISR), and the wealth of data it collected over the years, as a potentially transformative tool do address this issue.

In this paper, we report on a systematic survey of sudden electron density changes in the daytime ionosphere, and their possible connections with lightning and solar flares. This analysis is performed using vertical profiles of electron density data collected over the Arecibo Observatory (18.35$^\circ$ N, 66.75$^\circ$ W, −46.7$^\circ$ dip latitude), in Puerto Rico. The location chosen is ideal for our study because Arecibo has hosted for many years the most comprehensive suite of instruments to probe neutral and ionized atmosphere above the mesopause (including the ISR used here). Additionally, similarly to other places in the Caribbean region, Puerto Rico has a high occurrence of tropical storms accompanied by lightning. Despite these reasons, to the best of our knowledge, there is only one attempt to carry out such study, which took place more than two decades ago^20^, before the latest radar upgrades^21^. Showen and Slingeland^20^ performed a case-study-only type of investigation, which lead to inconclusive results. In this manuscript, we take a different route, and rather than performing another case study, we present a systematic survey of overlapping electron density, lightning location, and solar flare occurrence archival data. The analysis reveals that sudden electron density changes coincident with lightning are characteristically different than the ones coincident with solar flares. The former most often correspond to electron density depletions, while the latter to enhancements in electronic content. Both types of electron density changes are also, on average, distinguishable from the underlying variability of the lower ionosphere as seen by the ISR. Differences can be found not only in the D region, but also in the E region just above it.

## Results

**Figure 1:**
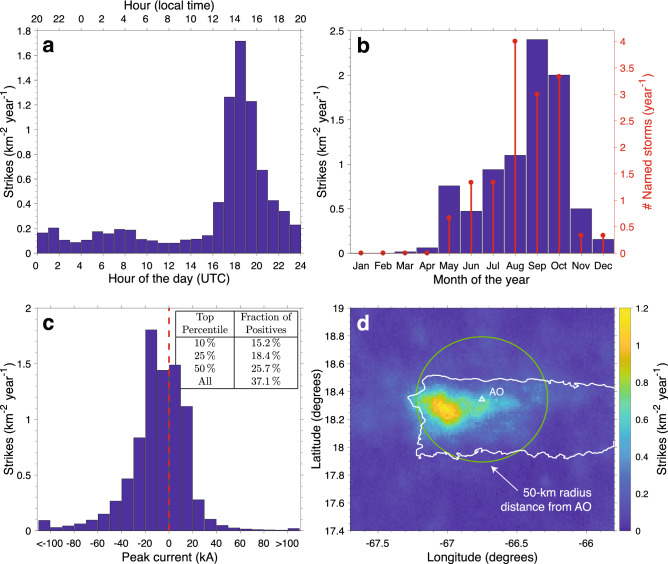
Lightning occurrence over Puerto Rico. Histograms of lightning occurrence rate per km$^2$ per year binned according to: (**a**) hour of the day, (**b**) month of the year, (**c**) peak current, and (**d**) geographical location. The data used covers a 3-year period between 2012 and 2014. Panel (**b**) also shows the number of named storms in the Atlantic ocean per year during the 2012–2014 period. The inset table in panel (**c**) shows the polarity breakdown by top percentile. Among the top 10% strongest flashes, 15.2% are of the positive polarity. Panel (**d**) also shows overlaid the Arecibo Observatory’s location (triangle), the outline of Puerto Rico’s main island, and a circle marking 50-km distance from AO for reference.

### Lightning occurrence rates over Puerto Rico

Figure 1 shows lightning occurrence rates around Puerto Rico’s main island for the 2012–2014 period colleted by Vaisala’s GLD360 detection network^22^ (in units of strikes per km$^2$ per year), binned according to hour of the day (Fig. 1a), month of the year (Fig. 1b), peak current (Fig. 1c), and geographical location (Fig. 1d) (see the Methods section for details). Figure 1a shows a clear peak at 17:00-20:00 UTC, or about 2:00 PM local time, while Fig. 1b identifies the *lightning season* taking place in the months of August, September, and October. For reference, Fig. 1b also shows the number of named convective systems in the Atlantic, Caribbean, and Gulf of Mexico, per month for the same 3-year period. This information is collected by the National Hurricane Center and is available online in the form of yearly reports^23–25^. The strong correlation between the two time series in Fig. 1b (with a Pearson correlation coefficient of 0.85) demonstrates a clear coincidence between the lightning and hurricane seasons in the region. It is worth mentioning that later in 2017 a storm such as the ones included in Fig. 1b, the infamous hurricane Maria, wreaked havoc in Puerto Rico, inflicting severe damage to the Arecibo Observatory and its incoherent scatter radar^26^. Strong winds associated with Hurricane Maria shattered the line feed^27^ used to collect the ionospheric data used in this investigation.

Figure 1c shows the distribution of peak currents. The median absolute value is 13 kA. The figure shows that a remarkably high fraction of all strikes detected by GLD360 in the region are of positive polarity (37%). It is widely known that positive cloud-to-ground (CG) flashes are more prone to initiate sprite discharges than negative ones^14,28–31^, therefore having a larger impact in the lower-ionosphere electron density than all other types of lightning flashes. The dataset used here includes both intracloud (IC) and CG flashes, but an attempt to isolate CG flashes can be made by looking only at the strongest discharges. The inset in Fig. 1c shows that among the top 10% strongest discharges, 15% are of the positive polarity. It also shows that among the top 25–50% strongest, a fraction of 18–26% are positives. These values are higher than most of the positive CG fractions reported in literature, which typically place this number at about or under 10%^32,33^.

Besides the apparent high proportion of $+$CG flashes, a second reason that makes a compelling case for our study is shown in Fig. 1d. We can see that most lightning activity is concentrated in a hotspot in the western part of La Cordillera Central mountain range. But more importantly, this hotspot is located only 30 km away from the Arecibo Observatory, which historically housed the most comprehensive suite of instruments on the planet for probing the ionospheric variability.

**Figure 2:**
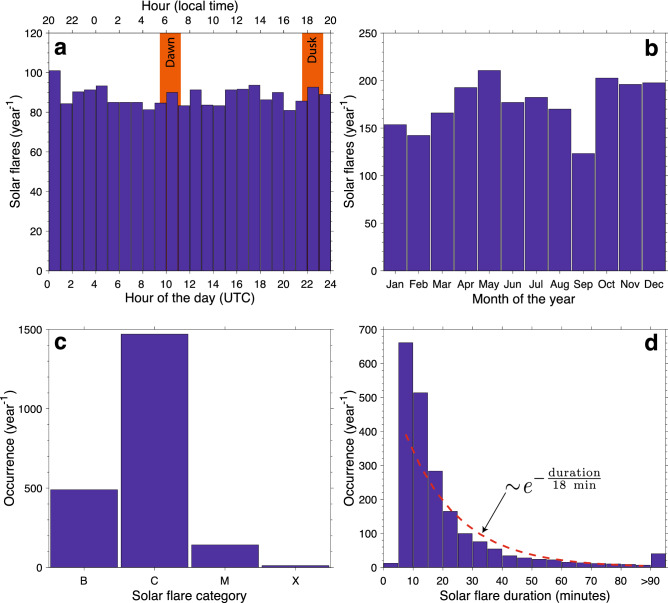
Solar flare occurrence rate in 2012–2014. Histograms of solar flare occurrence per year binned according to: (**a**) hour of the day, (**b**) month of the year, (**c**) category, and (**d**) duration.

### Solar flare occurrence rate

In order to clearly identify the effects of lightning in the daytime ionosphere, it is crucial to discriminate them against electron density changes due to solar flares, which predominantly impact the D region^7,8,34^. In the 3-year period of interest, between 2012 and 2014 (ascending phase of solar cycle #24), more than 2,000 solar flares were recorded each year, with roughly uniform occurrence rate as a function of hour of the day (Fig. 2a), and month of the year (Fig. 2b), and are predominantly of class C (Fig. 2c). Given the uniform probability distribution shown in Fig. 2a, it is easy to see that about 1,000 solar flares happened per year during local daytime in Puerto Rico, i.e., half of the total amount of solar flares reported took place from dawn until dusk, as marked in the figure. The median duration of solar flares in this period is 14 min, and it follows an exponential probability distribution (Fig. 2d), with an *e*-folding scale of 18 min. The median solar flare duration increases with its magnitude. More specifically, the median duration of a B, C, M, and X solar flare is 11, 14, 19, and 32 min, respectively.

### Sudden electron density changes in the daytime ionosphere (or spikes)

**Figure 3:**
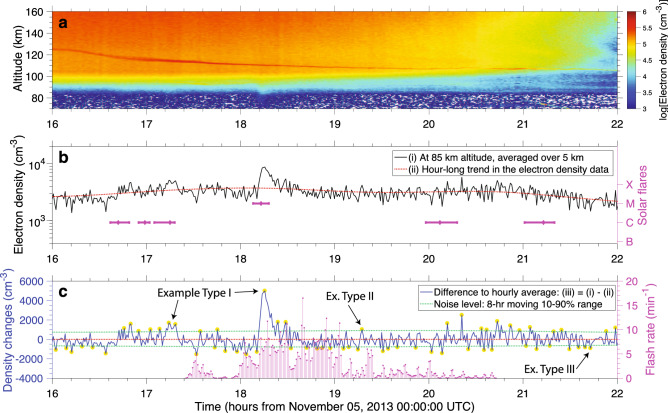
Ionospheric electron density recorded on November 05, 2013. (**a**) Logarithm of electron density data (in cm$^{-3}$) as a function of altitude (with resolution of 300 m) and time (with resolution of 50 s). (**b**) Electron density time series at 85 km altitude (left-hand-side vertical axis) and solar flare occurrence (duration and category, as shown in the right axis). Curve (i) shows data averaged over a ± 2.5 km altitude range, and curve (ii) shows the data further smoothed with an hour-long moving average. (**c**) Electron density changes (left axis), and flash rate in strikes/min (right axis). Curve (iii) is simply the difference between curves (i) and (ii). Panel (**c**) also shows the noise level (green dashed curve), defined as 10–90 percentile changes in an 8-h-long moving window. The electron density spikes referred to in the text are highlighted with yellow dots. Some examples of the three different types of spikes are labelled with text.

In this paper, we analyze altitude profiles of electron density recorded with Arecibo’s 430 MHz incoherent scatter radar^21,27^ (ISR) collected with 300-m spatial resolution. Figure 3a shows the electron density (in a logarithmic scale) as a function of altitude and time, recorded on November 05, 2013. Potential sudden electron density changes due to solar flares or lightning activity are swamped by the inherent variations with altitude and time, which span 3 orders of magnitude between 70 and 160 km altitude. Curves (i) to (iii) are designed to alleviate this problem. Curve (i) in Fig. 3b shows the electron density time series at 85 km altitude, averaged over a 5-km window around this altitude. Curve (ii) is an hour-long moving average of curve (i). Finally, taking the difference between the two curves, shown as curve (iii) in Fig. 3c, we are able to identify minute-long electron density changes at 85 km altitude as spikes in the time series (highlighted by yellow dots). We flag an electron density change as significant (i.e., store it for the subsequent systematic survey) if it is above the noise threshold, shown as dashed green curves in Fig. 3c. On the right-hand-side axis, Fig. 3b,c show the occurrence of solar flares and lightning, respectively. The flash rate nearby the Arecibo Observatory is given in strikes/min (see the Methods section for details).

It is easy to see from Fig. 3b,c that, despite the growing lightning activity nearby the Arecibo Observatory, the large electron density spike at 18:15 UTC is due to a class M solar flare. The ionization patch created between 77 and 87 km altitude amounts roughly to a doubling of the background electronic density, i.e., to a 5,000 cm$^{-3}$ increase. The electron density enhancement lasts for about 13 min, which is 4 min longer than the flare itself. Figure 3b shows that throughout the duration of the class M solar flare, the electron density increases from 4$\times$10$^3$ to 9$\times$10$^3$ cm$^{-3}$. As a point of comparison, the only other investigation looking into the solar flare effects in the D region using Arecibo’s ISR radar reported an increase in electron density at 85 km altitude from 7$\times$10$^3$ to 5$\times$10$^4$ cm$^{-3}$ due to a class X solar flare, which lasted for about 30 min^34^. Any potential electron density changes due to the coincident lightning activity are dwarfed by the class M solar flare. The other three preceding solar flares in the figure also increase the electronic content momentarily.

**Figure 4:**
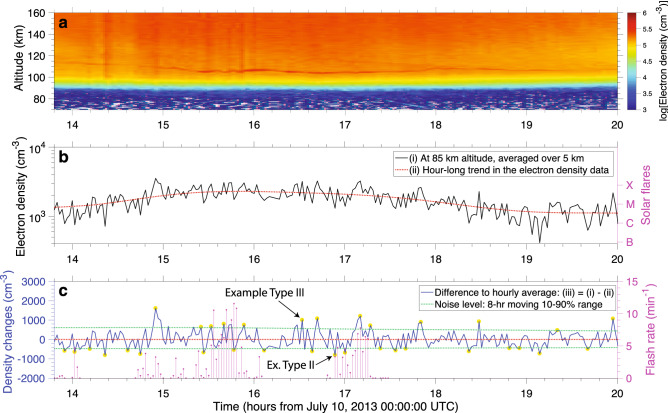
Ionospheric electron density recorded on July 10, 2013. Panels (**a**), (**b**), and (**c**) show the same information as in Fig. 3, but for a 6-hr period on July 10, 2013. Note from panel (**b**) that there are no solar flares during this time interval. The temporal resolution during this portion of the data is 100 s. Figs. 3c and 4c show examples of the spike classification discussed below as part of a systematic survey of the entire data set.

Figure 4 has the same format as Fig. 3, but displays electron density data recorded on July 10, 2013. There were no solar flares during the selected period, between 14:00 and 20:00 UTC. The two distinguishable spikes in Fig. 4c (at 14:55 and 17:10 UTC) are coincident with underlying lightning activity. These two ionization patches lasted for about 6–8 min, which means that if observed via VLF scattering, they would be classified as long recovery events or LOREs^15^. But more importantly, differently than solar flare-related changes, which tend to mostly create electron density enhancements, the lightning-related changes tend to most often be associated with electron density depletions^16^, as discussed below. It is evident from Figs. 3–4 that it is difficult to distinguish by eye electron density changes coincident with lightning from the ones attributed to solar flares, or simply due to the random fluctuation of the ionospheric density. Furthermore, a direct cross-correlation between the electron density change and lightning flash rate time series does not yield a clear correlation between the two. For these reasons, we resort to a systematic survey described below.

Another interesting feature in Figs. 3a and 4a is the presence of a sporadic E layer^35^, also referred to as a tidal ion layer (TIL) by several authors^36–38^, descending diagonally from 130 to about 100 km, which would be categorized as an upper semidiurnal trace in the nomenclature used by Christakis and colleagues^36^. In our data set we’ve found that in 3 of the 4 days for the July 8–12, 2013 period, a small but noticeable increase (of 10% of less) in the electronic content of the sporadic E layer happened in coincidence with underlying thunderstorms. One of the examples is the case shown in Fig. 4a, where a small increase in the sporadic layer (located between 102 and 107 km altitude) electronic content can be seen between 15:15 and 17:20 UTC. This finding is in agreement to the conclusions of Davis and Johnson^39^ that lightning causes an intensification of the sporadic E layer^40^. The behavior seen in Fig. 4a is only seen in the July, 2013 data, and it is not present in the data collected in November, 2013, and March, 2014 periods.

### Systematic survey results

We present a systematic survey of 316 hours of approximately continuous electron density data collection by Arecibo’s ISR distributed during three time periods on July/2013, November/2013, and March/2014 (see the Methods section for details). Within the 18,606 electron density profiles analyzed, we catalogued 212,045 sudden electron density changes, or spikes, amounting to an average of 1,030 per radar scan altitude level between 80 and 150 km in the daytime ionosphere. This is done by retrieving curve (iii) shown in Figs. 3 and 4 for all the 234 altitude scan levels between 80 and 150 km and isolating the spikes that emerge above the noise level (also defined in Figs. 3 and 4). Furthermore, we discriminate the spikes into 3 categories: the ones coincident with solar flares (type I, 11%), the ones coincident with lightning, but not solar flares (type II, 8%), and the ones not coincident with either, i.e., that are due to random fluctuation or due to other external factors (type III, 81%). In this study, we look into daytime data only, because it consists the vast majority of the disturbances found in our dataset (due to largest coincidence rate between availability of both lightning and ISR data), and because daytime electron density changes associated with lightning remain remarkably challenging to probe with other techniques than the one used here (such as VLF scattering and optical remote sensing). Besides discriminating the spikes regarding their potential source mechanisms, we also separate them into two altitude ranges: D region (between 80 and 100 km) and E region (100–150 km altitude).

**Figure 5:**
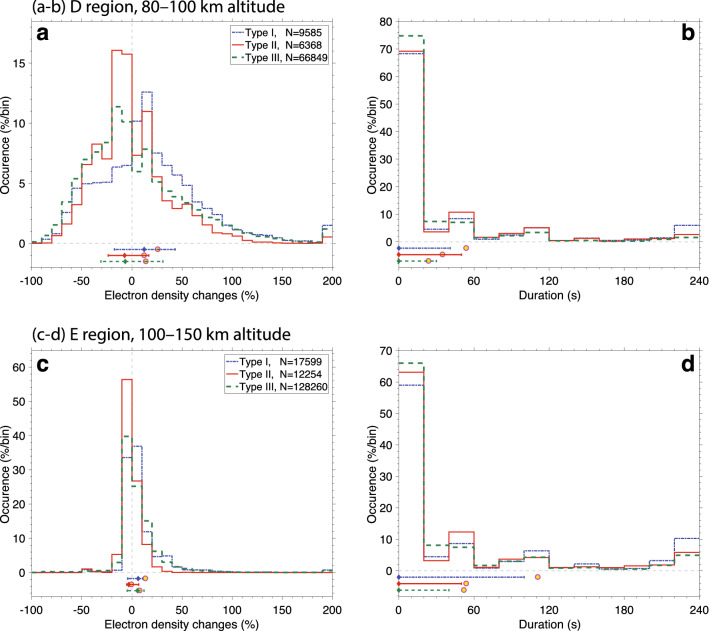
Statistical properties of sudden electron density changes, or spikes. Distributions of relative spike magnitudes (**a**,**c**) and durations (**b**,**d**), for two different altitude ranges, 80–100 km (**a**,**b**), and 100–150 km (**c**,**d**). The different curves in each panel show the different spike types, which are briefly defined in the bottom part of Table 1. The horizontal lines in the bottom part of each panel show the interquartile range of each data set, while the diamond and the circle show the median and average, respectively.

Figure 5 shows the distributions of the two most important spike properties: magnitude (Figs. 5a,c) and duration (Figs. 5b,d). The magnitude is given in relative terms, i.e., in % of the undisturbed level at the same altitude. This is calculated by taking the ratio of curves (iii) and (ii), shown in Figs. 3 and 4. The duration is defined from the number of subsequent radars scans that remain above the noise level. If a spike appears in a single electron density profile, its duration is counted as 0 s. To help visualize the difference in distribution shape between the three types of spikes, we add horizontal lines to the bottom of all four panels to mark the interquartile ranges. The overlaid diamonds and circles show the median and average, respectively. Summary statistics for the three types of spike distributions at the two altitude ranges of interest are presented in Table 1. In the bottom part of Table 1, we also provide a brief definition of the three spike types to facilitate the upcoming presentation of results.

The systematic survey performed here is essentially equivalent to a superposed epoch analysis. The electron density changes *due to* solar flares or lightning take place on top of the underlying variability of the ISR data, i.e., on top of the noise. However, when an average (or median for that matter) is taken over a large number of samples, meaningful features start to rise above the noise level. Bearing these statements in mind, the remainder paragraphs in this section highlight our key findings regarding type I (coincident with solar flares) and type II (coincident with lightning) spikes.

**Table 1:** Summary properties of sudden electron density changes (or spikes).

#	Occurrence	Region	Spike type		
			I	II	III
1	Spike sample size, N	D	9,585	6,368	66,849
		E	17,599	12,254	128,260
		Both	11,402	5,534	195,109
2	Fraction of entire data set (%)	Both	11	8	81
3	Average number of spikes per radar scan altitude level	D	143	95	998
		E	105	73	763
		Both	116	80	834
	**Relative spike magnitudes**				
4	Median (%)	D	$+$12.2 ± 0.6	−7.4 ± 0.5	−6.7 ± 0.3
		E	$+$6.2 ± 0.1	−3.2 ± 0.1	$+$5.5 ± 0.1
5	Difference to type III median (%)	D	$+$18.8 ± 0.7	−0.7 ± 0.6	–
		E	$+$0.7 ± 0.1	−8.7 ± 0.1	–
6	KS test statistic relative to type III	D	0.19	0.09	–
		E	0.10	0.20	–
	**Spike durations**				
7	Mean (s)	D	54 ± 3	35 ± 2	24 ± 1
		E	111 ± 5	54 ± 2	52 ± 1
8	Difference to type III mean (s)	D	30 ± 3	11 ± 2	–
		E	59 ± 5	2 ± 2	–
9	KS test statistic relative to type III	D	0.10	0.11	–
		E	0.12	0.09	–
	**Spike type definitions**				
**I**	Electron density spikes coincident with solar flares				
**II**	Spikes coincident with lightning, but not solar flares				
**III**	Spikes that are not coincident with either lightning or solar flares				

Figures 5a,b and Table 1 show that, in the D region, electron density changes coincident with solar flares (type I) have a larger median amplitude than the ones due to random chance (type III). More specifically, the difference in median amplitude is $+18.8\pm 0.7$%. On the other hand, the difference in median amplitude between spikes coincident with lightning (type II) and the ones due to random chance is found to not be significant, $-0.7\pm 0.6$%. A similar conclusion is found when looking at difference in mean spike durations, i.e., both type I and II have longer durations than type III, but the difference in mean duration is more significant in the first case ($30\pm 3$ s longer for type I and $11\pm 2$ s longer for type II). Next, we look into distinguishing the overall difference in the distributions shown in Figs. 5a,b. In this article, we measure the difference in distribution shape using the two-sided Kolmogorov–Smirnov test statistic (KS). This quantity is defined as the maximum difference between two cumulative distribution functions. A value of zero would indicate that the two datasets are sampled from the same probability distribution (the so-called null hypothesis). The maximum theoretical value of this quantity is 1, but 0.5 indicates a very large difference between two distributions. In the Methods section we perform simulations with synthetic data to demonstrate that small differences between two distributions map into KS values of $\le 0.03$ and that moderate (but easily identifiable) differences map into KS values of $\sim$0.2. Rows 6 and 9 of Table 1 show that only the distribution of type I electron density changes is noticeably different than random chance, with a KS value of 0.19. Looking at the actual Fig. 5a, we can see that the distribution of type I electron density changes has a distinct spike between $+\,10$ and $+\,20$% changes.

In the E region (Fig. 5c,d) the paradigm stated in the previous paragraph is shifted. In the E region, the electron density changes coincident with lightning (type II) have a noticeably lower relative amplitude than the ones due to random chance (type III). The difference in median amplitude is $-8.7\pm 0.1$%. This statement is supported by a KS value of 0.20. Looking at the actual distributions shown in Fig. 5c, we can see that type II spike distribution is narrower and has a sharp peak between $-\,10$ and 0%. On the other hand, the difference in amplitude for spikes coincident with solar flares is negligible $+0.7\pm 0.1$%. Similarly to the D region case, type I spikes have longer duration than type III (59 ± 5 s longer). The difference between type II and III durations is found to not be significant (only $2\pm 2$ s).

Generally speaking, in both D and E regions, type I spikes have larger median magnitude and average duration than type III. This fits the model that type I spikes are caused by a sudden increase in solar X-ray flux, penetrating to lower ionospheric altitudes, and enhancing the electronic content for several minutes. Please note that the durations reported in Figs. 5b,d and rows 7 and 8 of Table 1 are not the precise durations of the electron density disturbances. More precisely, the quantity reported here represents the number of subsequent electron density scans in which curve (iii) is above a stringent noise level, as shown in Figs. 3 and 4. For type I spikes the difference in median electron density change is more significant in the D region, while the difference in mean duration is more significant in the E region. One of the easiest examples to identify by eye in our data set is the event shown in Fig. 3 at 18:15 UTC on November 05, 2013, produced by a class M solar flare.

Differently than type I spikes, type II ones most often correspond to electron density depletions, i.e., they have negative median relative electron density changes (see row 4 in Table 1). These electron density changes typically appear in a single radar scan and, thus, have short durations (the average duration is only slightly longer than for type III spikes). A potential mechanism that can explain these findings is the penetration of weak lightning electric fields into the lower ionosphere driving electron attachment to oxygen molecules, as discussed by Shao and colleagues^16^. However, it remains unknown how lightning electric fields could penetrate into the E region. In our dataset, we surprisingly find that the most significant difference in median relative magnitude between type II and III spikes happens in the E region. This is due in part to the fact that there is a lower variability in the the electron density changes in the E region (compare the interquartile ranges in Fig. 5a,c).

## Summary and outlook

In this paper, we have presented a systematic survey of sudden electron density changes in the daytime lower ionosphere over the Arecibo Observatory, measured with an incoherent scatter radar. We discriminate the catalogued sudden electron density changes, or spikes, into three categories: (I) the ones coincident with solar flares, (II) the ones coincident with lightning, but not solar flares, and (III) the ones not coincident with either and assumed to be due to the underlying variability of the ionosphere. The key takeaway message from this systematic survey is that the three types of spikes are different in nature, but the identified differences are small to moderate in magnitude. Sudden electron density changes coincident with solar flares most often produce ionization, while the ones coincident with lightning most often cause electron density depletions. Both types of sudden electron density changes have longer duration than the underlying fluctuations present in the data, but only in the case of electron density changes coincident with solar flares, the longer duration is significant.

This investigation opens a pathway for using Arecibo’s ISR, and the wealth of data it has acquired over the years, as a transformative tool to quantify the effects of lightning in the lower ionosphere. At the time of writing of this paper, the suspended instrument platform collapsed on the Arecibo radio telescope dish on December 1, 2020^41^. The collapse followed the failure of two main cables^42^ that held the instrument platform. The cable failures started in the second semester of 2020, after years of wear and tear, and after having survived the damage from thunderstorms systems^26^ like the ones studied in this paper. Investigations such as this one show that the wealth of data collected over the years by the Arecibo Observatory will continue to fuel scientific discoveries well beyond the life cycle of the radio telescope.

## Methods

### Lightning location data

We use geolocation and peak current information provided by Vaisala’s GLD360 global lightning detection network. GLD360 has a location accuracy of the order of 5 km and 50–70% detection efficiency for cloud-to-ground (CG) lightning strikes^22^. In this analysis, we use lightning location data for a 3-year period between 2012 and 2014 (ascending phase of solar cycle #24), covering a region that spans 200 km in longitude ($-67.7^\circ$ W to $-65.8^\circ$W) and 178 km in latitude (17.4$^\circ$N to 19.0$^\circ$N) approximately centered on the Arecibo Observatory. During this time period, GLD360 did not discriminate between intracloud (IC) and CG lightning strikes. Therefore the dataset includes both types, but the detection system favors the strongest CG strikes^22^. One lightning flash may contain multiple lightning strikes. For the sake of completeness, we use individual strikes in the analysis, and do not group adjacent strikes into flashes, since individual strikes (of both IC and CG flashes) can impact the ionosphere^43^.

The flash rate shown in Figs. 3c and 4c is determined from a custom 3-step process. First, we select only lightning strikes that are within 50 km of the observatory (green circle in Fig.  1d). Second, we correct for peak current, normalizing all discharges to 13 kA, the median amplitude in the dataset. Third, we bin the flash rate in a histogram that is temporally aligned to the electron density profiles’ time stamp (in 1 min bins). The normalization to peak current ensures proper weighting of the potential ionospheric impacts of a lightning strike, as the the radiated electromagnetic field is proportional to this quantity^44^. Additionally, the 50-km threshold distance ensures that not only the radiative, but also the electrostatic component of the lightning electric field can reach the ionosphere over the ISR radar^44^.

### Solar flare data

In this paper, we use the GOES X-ray solar flare catalog archived by NOAA at www.ngdc.noaa.gov/stp/solar/solar-features.html. We use information on solar flare onset time, duration, and magnitude (i.e., its class) for the period of 2012–2014 (ascending phase of solar cycle #24), as shown in Fig. 2. In Fig. 3b we show the duration of each solar flare as a horizontal bar, and the instant of peak emission as an overlaid diamond. Solar flares are classified into four different categories based on their peak emission in the 0.1–0.8 nm X-ray range: A (>10$^{-7}$ W/m$^2$), B (>10$^{-6}$ W/m$^2$), C (>10$^{-5}$ W/m$^2$), and X (>10$^{-4}$ W/m$^2$). These categories are shown in Figs. 2c and 3b. A total of 103 solar flares took place during the three periods investigated.

### Ionospheric electron density data

We analyze vertical profiles of electron density probed with Arecibo’s 430 MHz incoherent scatter radar^21,27^ (ISR). The data is freely available from the Madrigal database. We look into three periods of approximately continuous, high-spatial-resolution (300 m) data collection by the Arecibo ISR. The three periods are: 99 h starting on July 08, 2013 13:42 UTC, 69 hours starting on November 04, 2013 19:49 UTC, and 148 h starting on March 31, 2014 13:17 UTC. The 18,608 electron density profiles analyzed were not equally spaced in time. Typical intervals were also different among the three periods analyzed. More precisely, the time intervals ranged between: 10–100 s (for the July/2013 period), 10–50 s (November/2013), and 10–60 s (March/2014). Besides the three periods analyzed, there are additional periods with available ISR electron density data archived at the CEDAR Madrigal database during the years of 2012 to 2014. However, they have not been selected for this study due to the lack of overlap with GLD360 lightning-location data.

The technique introduced here to identify sudden electron density changes allows us to perform a systematic survey in our dataset and to contrast, in a statistical sense, the spikes that are associated with lightning activity to the ones that are not. However, it is evident from Fig. 3a that the technique has some limitations. For instance, we can see a diagonal trace flagged at about 21:00 UTC below 90 km altitude. This trace is the echo of a ship on the ocean passing nearby the island.

Please also note that the electron density data undergoes substantial processing before being deposited in the CEDAR Madrigal database to remove any type of interference not already removed by software or hardware during collection. One could argue that the lightning-related electron density changes reported here are due to lightning electromagnetic interference. However, there are two key factors that indicate otherwise: (i) lightning high-frequency emissions last a small fraction of a second, while the integration period of the Arecibo radar varies between several seconds to minutes, depending on the desired application, and (ii) these electron density changes registered in coincidence with lightning are most often *depletions* and not enhancements (as how one could expect VHF noise to contaminate the measurements).

### The Kolmogorov–Smirnov test statistic

**Figure 6:**
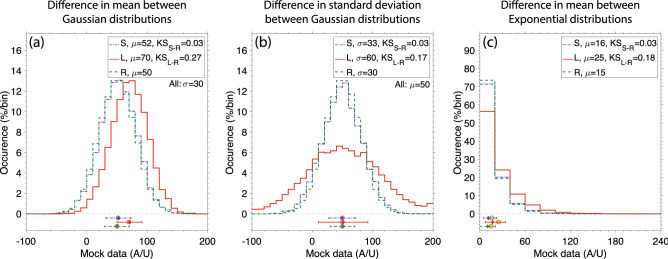
Illustrating the Kolmogorov–Smirnov test statistic (KS). Simulations with synthetic data generated according to Gaussian (**a**,**b**) and exponential (**c**) probability distributions. The results are present in a similar manner as Fig. 5. Each panel shows a reference case (R), and two additional cases with a large (L) and small (S) change in mean $\mu$ (**a**,**c**) and standard deviation $\sigma$ (b). The resulting KS values are shown in the figure legend. In all panels, the R population has a size of 60,000, while the S and L populations have a smaller population size of 6,000. This is done to roughly match the difference in population sizes in Fig. 5.

The two-sided Kolmogorov–Smirnov test statistic (KS) is defined as the maximum difference between the cumulative probability distribution functions of two different populations^45^. While the KS value may vary between 0 and 1, a value as low as 0.2 can indicate a clear/distinguishable difference between two distributions. In order to illustrate that, we perform simulations using synthetic data drawn from Gaussian (Fig. 6a,b) and Exponential (Fig. 6c) probability distributions. The green line in Fig. 6a is the reference case (R) with mean $\mu$ = 50 and standard deviation $\sigma$ = 30. The mock data are dimensionless, but have numerical values similar to the electron density changes (Fig. 6a,b) and durations (Fig. 6c) reported in Fig. 5. For comparison the figure shows two other cases with small (S) and large (L) changes in mean to $\mu$ = 52 and 70, respectively. We can see that the S case has a KS value of 0.03, while the L case yields a KS value of 0.27, both with respect to case R. We repeat the analysis in Fig. 6b to show how small (S) and large (L) changes in $\sigma$ map into KS values, the effort yields similar conclusions (with KS equal to 0.03 and 0.17 for small and large changes in $\sigma$, respectively). Finally, the analysis is repeated once again for an Exponential probability distribution, which better aligns with the distribution of durations shown in Fig. 5. note that for an Exponential distribution the average and the standard deviation are equal to each other, and are representative of the *e*-folding scale of the distribution tail. Once again, small changes in $\mu$ map into KS = 0.03, while large changes map into KS = 0.18. The simulations performed here give us confidence to state that the KS values reported in Table 1, of $\sim 0.2$, correspond to real differences between the three types of electron density distribution shown in Fig. 5.

All of the average, median properties, and respective differences in Table 1 are accompanied by an uncertainty estimate defined as the corresponding 95% confidence interval. For normally-distributed data, the 95% confidence interval around the mean ($\mu$) is derived from the Student’s *t*-test, and is equal to 1.96$\sigma /\sqrt{N}$, where $\sigma$ is the population standard deviation and *N* is the total number of observations. If the data does not follow a normal distribution, the 95% confidence interval can be obtained for nonparametric data using fractional order statistics and bootstrapping^46,47^. Similarly, the two-sided *t*-test and fractional order statistics^46,47^ are used to calculate the differences in mean and median, respectively (as reported on rows 5 and 8 of Table 1).

## Data Availability

Archival Arecibo ISR electron density data is available at the cedar.openmadrigal.org database. GOES X-ray solar flare data is available at www.ngdc.noaa.gov/stp/solar/solar-features.html. Contact Vaisala Inc. directly for access to lightning data.
